# Synthesis and Characterization of Lignocellulose-Based Carbon Quantum Dots (CQDs) and Their Antimicrobial and Antioxidant Functionalities

**DOI:** 10.3390/molecules30030667

**Published:** 2025-02-03

**Authors:** Wooseok Lee, Seonghyuk Ko

**Affiliations:** Laboratory of Nano-Enabled Packaging & Safety, Department of Packaging, Yonsei University, Wonju-si 26493, Republic of Korea; dltjr123@nate.com

**Keywords:** carbon quantum dots, lignocellulose, hydrothermal synthesis, antimicrobial property, antioxidant activity

## Abstract

Carbon quantum dots (CQDs) have recently drawn enormous attention due to not only their unique chemical, biological, and optical properties but also because a variety of renewable biomasses are readily utilized as carbon sources in their synthesis. This study investigated the synthesis, characterization, and functional evaluation of CQDs from unbleached mechanical pulp as a natural lignocellulosic resource. The CQDs were synthesized using a one-step hydrothermal synthesis with varying temperature, time, and pulp consistency. The resulting CQDs exhibit a spherical shape with a size distribution of 9.73 ± 0.82 nm and lattice parameters of 0.21 and 0.34 nm, indicating a graphite core. The photoluminescence spectra showed evident fluorescence characteristics, with an emission peak at 435 nm at an excitation wavelength of 370 nm. The as-prepared CQDs were also chemically composed of C=C and C=O bonds linked to the hydroxyl and carboxyl functional groups, which are typically found in lignocellulose-based CQDs. The CQDs demonstrated antibacterial activity exceeding 99.9% against *E. coli* at the lowest concentration of 0.75 mg/mL. Demonstrating its antioxidation property, the DPPH radical scavenging activity surpassed 90% with more than 40 µg/mL of the CQD solution.

## 1. Introduction

Quantum dots (QDs) are novel fluorescent nanomaterials comprising inorganic cores linked to organic molecules that are typically 1 to 10 nm in size [1]. Conventional QDs are constructed using inorganic materials and heavy metals, including cadmium selenide (CdSe), zinc sulfide (ZnS), and indium phosphate (InP). Unlike conventional QDs, CQDs have a carbon core linked to additional nitrogen- or oxygen-rich surface functional groups [2,3]. Their diverse and unique features, including optical properties, biocompatibility, low toxicity, and antimicrobial and antioxidant properties, make them suitable for various applications, including bioimaging [4], heavy metal detection [5], drug delivery [6], and active/intelligent packaging [7,8].

CQDs are, as one of the emerging active substances, of particular interest in antimicrobial and antioxidant applications. Hao, et al. [9] reported the extensive antibacterial activity of positively charged CQDs against common bacteria and drug-resistant bacteria. In an infected wound rat model, the healing effect was quite comparable to that of levofloxacin. Akash, et al. [10] synthesized CQDs using sugarcane bagasse and presented their strong antioxidant activity of up to 77%, which is similar to that of ascorbic acid. It is a highly capable substitute for conventional nanomaterials and natural compounds such as silver nanoparticles, ascorbic acid, and tocopherol in antibacterial and antioxidation applications [11]. 

CQD synthesis methods can be classified as either top-down or bottom-up. The top-down method involves breaking down large carbon materials, such as graphite, graphene, and activated carbon, whereas the bottom-up method focuses on carbonizing small organic molecules or biomass [12,13]. The bottom-up method, which includes pyrolysis, plasma treatment, and hydrothermal synthesis, is preferred for its simplicity, cost efficiency, energy efficiency, and mass productivity [14]. Specifically, the hydrothermal synthesis method can easily reach the subcritical temperature between 180 and 250 °C for carbonization using self-generated pressure [1]. Moreover, the properties of CQDs can be easily adjusted by changing the material, temperature, and synthesis time [15].

Various types of biomass, such as wood residue, agricultural waste, plant extracts, fruit peels, and milk, have recently been examined as carbon sources for CQDs [8]. Lignocellulose, comprising cellulose, hemicellulose, and lignin, provides aromatic rings and ring structures with oxygen-based functional groups, making it an efficient carbon source for CQDs [16,17]. Lignocellulosic biomass is an abundant renewable resource [18,19]. One type of lignocellulosic biomass is the forest residue waste obtained from forest operations, such as leaves, wood chips, sawdust, stems, and branches, which account for 50% of the produced wood [20]. The International Energy Agency projected that global forest residues will increase to 0.7 Gt by 2030 [21]. Despite these several benefits, few studies have focused on utilizing forest biomass as a lignocellulose-rich natural source for high-value-added CQDs.

This study aimed to synthesize and characterize lignocellulose-based CQDs using unbleached mechanical pulp consisting of constituents similar to forest resources and to evaluate their antimicrobial and antioxidant properties for further utilization of lignocellulosic forest biomass. The syntheses were conducted using a one-step hydrothermal method, and the optimum conditions were suggested to obtain a high yield of CQDs. The chemical states and morphologies of the prepared CQDs were analyzed, and their antibacterial and antioxidant functions were investigated.

## 2. Results and Discussion

**Figure 1:**
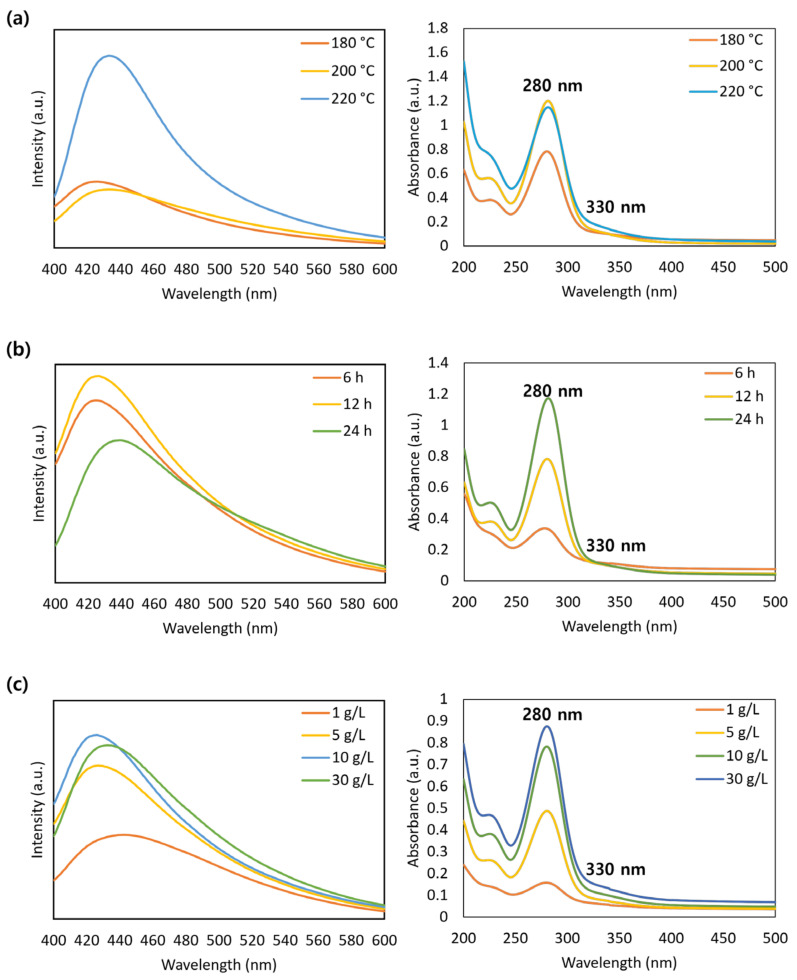
PL and UV–vis spectra of CQDs synthesized with variable temperature, time, and mass fraction conditions. (**a**) Temperature (at 12 h and 10 g/L): 180, 200, and 220 °C. (**b**) Time (at 180 °C and 10 g/L): 6, 12, and 24 h. (**c**) Mass fraction (at 180 °C and 12 h): 1, 5, 10, and 30 g/L.

Figure 1 presents the effect of the reaction parameters, e.g., temperature, time, and pulp mass fraction, on CQD synthesis and identifies the optimum conditions for obtaining higher yield. Figure 1a shows the photoluminescence (PL) and UV–Vis spectra at different reaction temperatures with a reaction time of 12 h and a mass fraction of 10 g/L. As the synthesis temperature is increased from 160 to 220 °C, the fluorescence intensity is also strengthened. In the UV–Vis spectra, the characteristic peaks of CQDs were identified as the π–π* transition of the C=C bond and n–π* transition of the C=O groups at 280 and 330 nm, respectively [22]. The hydrothermal synthesis of CQDs using lignocellulosic materials involves chain breaking, condensation, and formation [23]. Among the lignocellulose components, cellulose and hemicellulose are hydrolyzed at a relatively low temperature of 160 °C owing to their simple monosaccharide straight-chain structure with glycosidic bonds. In contrast, lignin, which has a complex three-dimensional structure composed of phenylpropan, only begins to hydrolyze at higher temperatures [15,24]. Consequently, increasing the reaction temperature is an effective way to achieve efficient CQD production when lignocellulosic materials are used as the carbon source.

Figure 1b presents the PL and UV–vis spectra of the CQDs according to the variable reaction times with a temperature of 180 °C and mass fraction of 10 g/L. The fluorescence intensity was highest at 12 h throughout the synthesis. Some studies have reported that longer reaction times enhance the fluorescence of CQDs [25,26]. However, Wu et al. [27] reported that excessively long reaction times decreased the yield of CQD production from biomass due to carbonization and the formation of large insoluble particles. Meanwhile, the UV–vis spectra showed that the peak at 280 nm increased with reaction time, which is thought to be due to the increase in C=C bonds, indicating that the carbon core grew proportionally to the time factor during synthesis [28].

The PL and UV–vis analysis results, in accordance with different mass fractions at 180 °C and 12 h, are shown in Figure 1c. The highest fluorescence emission was observed at a mass fraction of 10 g/L. The water-to-substance ratio is an essential parameter for hydrothermal reactions because a low initial concentration is insufficient for the condensation of carbonaceous materials, whereas a high concentration may cause incomplete hydrolysis at the beginning of the chemical reaction [29]. Therefore, an appropriate ratio of carbon sources to distilled water must be used. As a result, the optimal synthesis conditions in the present study were determined to be 220 °C, 12 h, and 10 g/L for the reaction time, temperature, and mass fraction, respectively.

**Figure 2:**
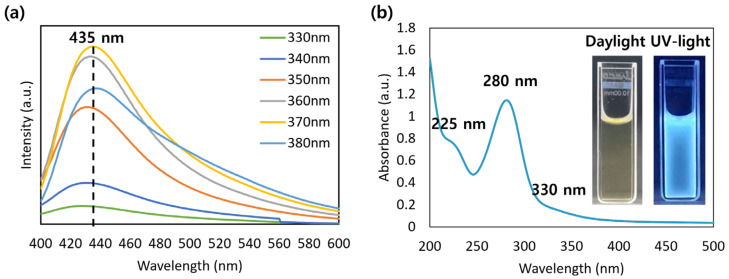
Optical characteristics of CQDs synthesized under the optimal synthesis condition. (**a**) PL emission spectra, (**b**) UV–vis absorption spectrum. Inset: CQD solutions under daylight and UV light.

Figure 2a shows the PL spectra of the CQDs prepared under optimal synthesis conditions. The fabricated CQDs emitted blue-green PL with an optimal excitation wavelength of 370 nm, and a redshift in the emission peaks occurred as the excitation wavelength increased [1]. The UV–vis spectrum of the CQDs is shown in Figure 2b. The peaks at 280 and 330 nm are attributed to the C=C and C=O bonds of the CQDs, respectively, which may be due to the aromatic polymer structure of the CQD and functional groups, such as hydroxyl and carbonyl groups, on the CQD [23]. The inset of Figure 2b shows a CQD solution emitting blue fluorescent light under UV light. Oxidization and oxygen-based functional groups on the CQD surface can cause surface defects, reducing the energy gap between the highest and lowest occupied molecular orbitals, which can generate fluorescence [30].

**Figure 3:**
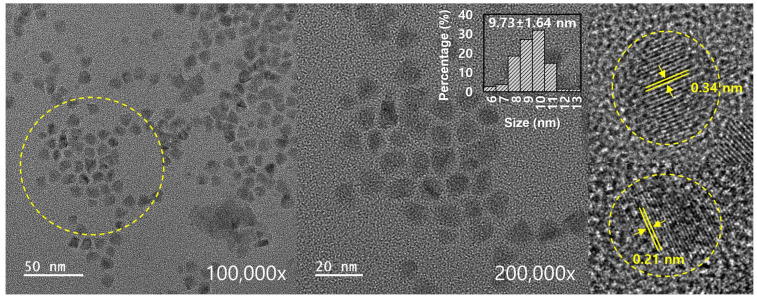
TEM images and particle size distribution.

The TEM images in Figure 3 show that the CQDs were uniform and spherical. Approximately 82 CQD particles were captured for analyzing the size distribution using Image J software, and it was found that they have a good distribution with an average size of 9.73 ± 1.64 nm. On the other hand, uniform and sharp lattice planes with d-spacings of 0.21 and 0.34 nm were also detected, reflecting the (100) and (002) diffraction planes of graphitic carbon, respectively [31,32].

**Figure 4:**
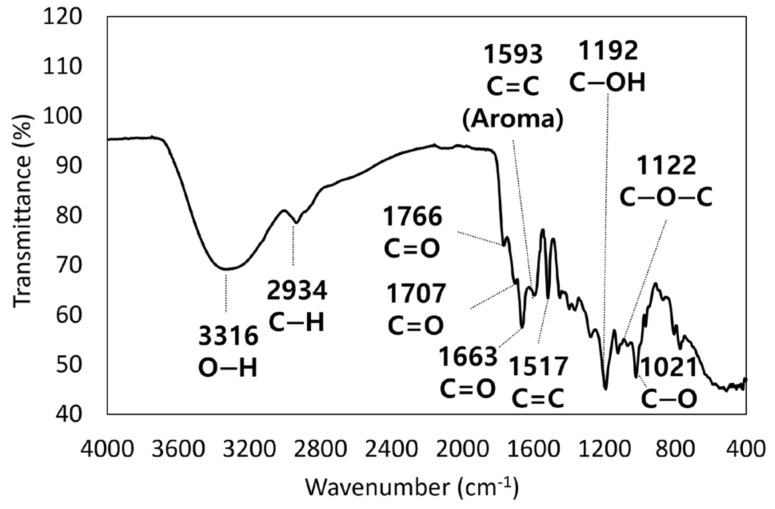
FTIR spectrum of CQD.

Figure 4 presents the FTIR spectrum of the freeze-dried CQD powder. The broad peaks at 3316 and 2934 cm^−1^ are related to the stretching vibration of O-H [33] and the bending vibration of C-H [34], respectively. This may be due to the moisture and carbohydrate contents of the raw material. The peaks found at 1593 and 1517 cm^−1^ could be correlated with the sp^2^ structure of the C=C bond and aromatic structure [23,35]. These results indicate that the fabricated CQDs possess an aromatic polymer structure featuring carbon double bonds, which correspond to the carbon peaks observed in the UV–vis spectra and the diffraction planes identified in the TEM results. Meanwhile, the absorption peak at 1122 cm^−1^ was attributed to the C-O-C bond, which generally appears in the lignin structure [35]. IR absorption peaks associated with the carbonyl group (C=O) were also observed at 1760, 1707, and 1663 cm^−1^ [36,37,38]. The signals observed at 1021 and 1192 cm^−1^ were coupled with the stretching of the C–O and C-OH bonds [39], indicating hydroxyl (OH) and carboxyl (COOH) groups attached to the CQDs [40].

**Figure 5:**
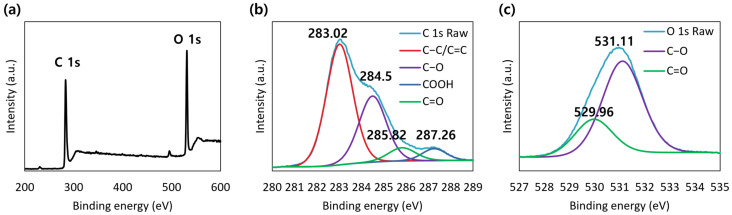
XPS spectra of CQD powder: (**a**) full-scan spectrum; high-resolution spectra of (**b**) C 1s and (**c**) O 1s.

Figure 5a displays the XPS full-scan spectrum of the CQD powder. Two prominent peaks at 283.87 and 531.59 eV associated with C and O were detected in the spectrum. However, the N 1s peak located at approximately 401 eV was not identified. High-resolution C 1s and O 1s XPS spectra of the CQDs are shown in Figure 5b and Figure 5c, respectively. The high-resolution C 1s spectrum exhibited four distinct peaks at 283.0, 284.5, 285.8, and 287.3 eV, corresponding to C-C/C=C, C-O, C=O, and COOH bonds [22]. Two different bonding states of oxygen were observed: C=O at 530.0 eV and C-O at 531.1 eV in the high-resolution O 1s spectrum [38]. These results indicate that the carbon within the CQDs is predominantly composed of graphitic carbon with oxygen-containing functional groups such as carbonyl and carboxyl groups [41].

**Figure 6:**
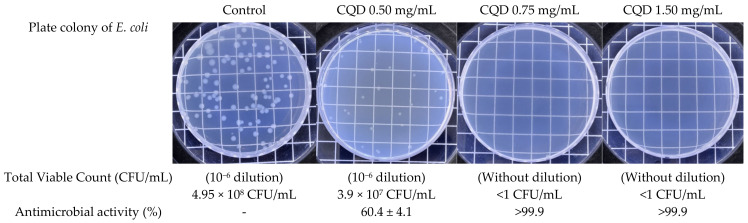
Antibacterial activity of CQDs with different concentrations (0.5, 0.75, and 1.5 mg/mL).

The results of the antibacterial tests on *E. coli* with various concentrations of the CQD solution are presented in Figure 6. The total viable count measured at a CQD concentration of 0.5 mg/mL was 3.9 ± 0.2 × 10^7^ CFU/mL, and its antimicrobial activity was calculated as 60.4 ± 4.1%, whereas no bacteria were found above a 0.75 mg/mL CQD concentration. Similar to many other antibacterial agents, CQDs exhibit concentration-dependent antibacterial activities [42]. Ma et al. [43] prepared CQDs using various biomass precursors and showed strong antibacterial reactions against *E. coli* and *Staphylococcus aureus* when the concentration of the CQDs increased to 1000 μg/mL. Yu et al. [40] reported that the antibacterial activity against *E. coli* was associated with the CQD content and time. A significant bacterial count reduction was observed for CQD solutions at concentrations exceeding 1 mg/mL. CQDs can attach to cell walls through diffusion and electrostatic interactions, which interfere with nutrient consumption and metabolic processes and lead to the disruption of the cell wall and the structure of nucleic acids [40]. The antibacterial properties of CQDs are attributed to their electrostatic properties resulting from their nanoscale size and carbon core bound to functional groups [1,12], as confirmed by the FTIR, UV-Vis spectrum, and TEM results.

**Figure 7:**
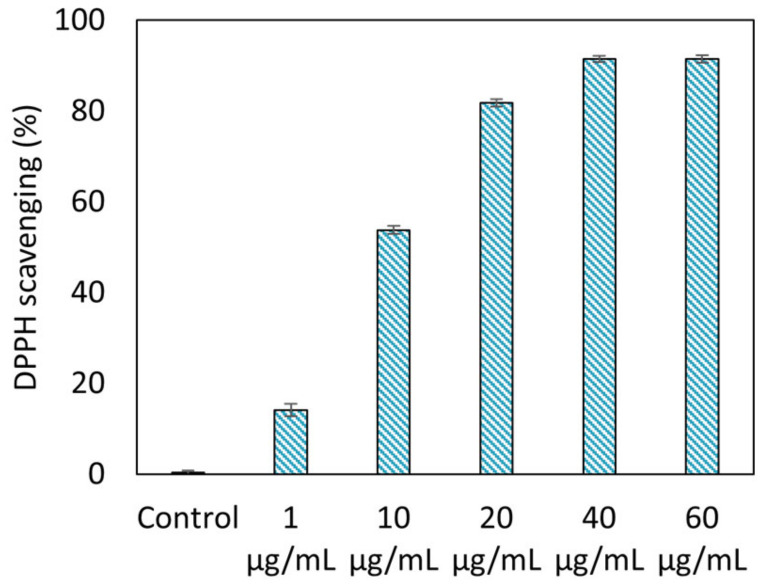
Antioxidant activity of CQDs with different concentrations (1, 10, 20, 40, and 60 μg/mL).

Figure 7 shows the DPPH antioxidant test results for various concentrations of CQD solutions. The radical-scavenging effect increased proportionally with the CQD concentration. The DPPH scavenging rates at CQD concentrations of 1, 10, and 20 μg/mL were 14.2 ± 1.4, 53.8 ± 0.8, and 81.8 ± 0.8%, respectively. At concentrations greater than 40 µg/mL, the scavenging effect on DPPH readily reached 91.5 ± 0.7% in 30 min. Many studies have reported the antioxidant properties of CQDs based on their DPPH scavenging power [40,44,45]. CQDs have antioxidant effects, as the hydroxyl and carboxyl groups on the surface donate hydrogen to DPPH, and the sp2 carbon network structure, such as fullerene, carbon nanotubes, and graphene, delocalizes and stabilizes free electrons [1,46].

## 3. Materials and Methods

### 3.1. Materials

The unbleached mechanical pulp was provided by Jeonju Paper (Jeonju, Republic of Korea). Distilled water was prepared using the Human Power I+ water purification system (Human Co., Seoul, Republic of Korea). Plate count agar (PCA) was purchased from BD Biosciences (Becton, Dickinson and Company, Franklin Lakes, NJ, USA). Muller–Hinton broth (MHB) was obtained from Sigma–Aldrich (Schnelldorf, Germany). 2,2-Diphenyl-1-picrylhydrazyl (DPPH) was obtained from Thermo Fisher Scientific (Waltham, MA, USA).

### 3.2. Synthesis of CQDs

The CQDs were prepared from mechanical pulp using a conventional hydrothermal synthesis method. First, the desired amounts of pulp and 300 mL of distilled water were mixed and rigorously stirred at 500 rpm for 60 min using an MS 3060D Mechanical Agitator (M-TOPS, Seoul, Republic of Korea) with a 30 mm diameter impeller. The mixed suspension was placed in a stainless-steel reactor with a 500 mL Teflon liner and heated in a dry oven at a specific temperature and time. Three separate temperature conditions, 180, 200, and 220 °C, were chosen with different reaction times: 6, 12, and 24 h. The pulp mass fractions used for the synthesis were 1, 5, 10, and 30 g/L.

After the synthesis under each condition, the reactor was left in ambient air for 60 min and then cooled to 25 °C using a water bath. The resultant sample was centrifuged at 4000 rpm for 10 min using a Labogene 406 centrifuge (Lillerød, Denmark) and filtered using a 0.22 μm syringe filter to eliminate insoluble matter. Subsequently, the resulting sample was centrifuged at 20,000 rpm for 20 min using a VELOSPIN 22R centrifuge (CRYSTE Korea, Gwangmyeong, Republic of Korea) and filtered using a 0.1 μm microporous filter membrane. Then, the as-synthesized CQD solutions were kept in a refrigerator (4 ± 1 °C) for further experiments. The concentration of the produced CQD solution was 1.5 mg/mL, which was determined by measuring the weight of CQDs present in 30 mL of solution obtained after freeze-drying for 2 d.

### 3.3. Characterization

The fluorescence properties of the CQDs were investigated using an RF-6000 spectrofluorophotometer (Shimadzu, Kyoto, Japan) at excitation and emission wavelengths in the range of 300–600 nm.

The optical characteristics of the CQDs were verified by ultraviolet–visible (UV-Vis) spectroscopy using a double-beam V-600 spectrophotometer (Jasco, Tokyo, Japan) with an attached 6-cell accessory (Jasco, Tokyo, Japan) in the wavelength range of 200–800 nm.

The microstructures and sizes of the CQDs were observed using a JEM-ARM 200F transmission electron microscope (TEM, JEOL, Tokyo, Japan) operating at 300 kV with a magnification of 500,000×. Prior to the examination, the concentrated CQD solution was dried on a copper grid (01814G-F, 400 mesh, TED PELLA Inc., Redding, CA, USA) at 50 °C. Finally, the TEM image was analyzed using ImageJ^®^ software version 1.54 m.

The Fourier-transform infrared (FTIR) spectra of the freeze-dried CQD powders were obtained using a PerkinElmer Spectrum 65 spectrophotometer (PerkinElmer Co., Ltd., Waltham, MA, USA) in the attenuated total reflection (ATR) mode with a C/ZnSe crystal. Each spectrum was obtained in transmittance mode with eight scans and a resolution of 2 cm^−1^ in the wavenumber range of 400–4000 cm^−1^.

The chemical composition and content on the surface of the CQDs were analyzed using a K-alpha+ X-ray photoelectron spectroscope (XPS, Thermo Fisher Scientific Inc., E. Grinstead, UK) employing an Al Kα µ-focused monochromator (1486.6 eV). The full-scan XPS spectra were scanned first, followed by narrow scans of the C 1s and O 1s spectra.

All characterizations were performed with three replicas from each synthesis.

### 3.4. Antimicrobial Property

The antimicrobial assay was conducted according to the method described by Yu et al. [40]. First, the *Escherichia coli* (*E. coli*, ATCC 8739) suspension concentration was maintained at 10^6^ CFU/mL in MHB medium. Subsequently, 1 mL of the bacterial dilution and 1 mL of various concentrations (0.5, 0.75, and 1.5 g/mL) of CQD solutions were mixed and cultured in an incubator at 37 °C for 24 h. Each solution was serially diluted 10 times in saline and incubated on PCA at 37 °C for 24 h to determine the total viable count. This experiment was carried out in triplicate, and the antimicrobial activity was calculated as the following equation:

(1) $$\mathrm{Antimicrobial}\;\mathrm{activity}\;=1-\frac{{\mathrm{CFU}}_{\mathrm{control}}-{\mathrm{CFU}}_{\mathrm{CQD}}}{{\mathrm{CFU}}_{\mathrm{control}}}\;\times 100\left(\%\right)$$

where CFU_control_ and CFU_CQD_ are the total viable count in the control and test samples, respectively, after 24 h.

### 3.5. Antioxidant Activity

The pristine CQD solution was subjected to DPPH radical scavenging tests according to the method described by Zhao et al. [39]. The CQD solution (2 mL) and deionized water as a control sample were separately mixed with 2.0 mL of a dark purple DPPH solution (100 μM in absolute ethanol) in a conical tube. The tubes were placed on a twist shaker (TW3, FINEPCR, Republic of Korea) and constantly shaken at ambient temperature for 30 min. Three samples were prepared under each set of conditions. The radical-scavenging activity was assessed by measuring the absorbance of the solutions at 517 nm using UV–vis spectroscopy. The average value was calculated by repeating the experiment five times. The DPPH-scavenging rate was calculated using the following equation:

(2) $$\mathrm{DPPH}\mathrm{Scavenging}=1-\frac{A_{\mathrm{after}}-A_{\mathrm{base}}}{A_{\mathrm{control}}}\;\times 100\left(\%\right)$$

where A_after_ is the absorbance after the reaction of the CQD solution and the DPPH ethanol solution, A_base_ is the absorbance after the reaction of the CQD solution and absolute ethanol, and A_control_ is the absorbance after the reaction of deionized water and the DPPH solution.

## 4. Conclusions

In this study, CQDs were prepared via hydrothermal synthesis using unbleached mechanical pulp as a model lignocellulosic byproduct, and their antibacterial and antioxidant properties were characterized and evaluated. The PL spectra revealed that the as-prepared CQDs emitted blue–green fluorescent light when excited by UV light at wavelengths of 330–380 nm with an optimal excitation wavelength of 370 nm. This phenomenon may be attributed to surface defects and functional groups, that is, hydroxyl and carboxyl groups, as confirmed by the UV–vis, FTIR, and XPS spectra. The TEM results illustrated that CQDs have a graphite carbon core that measures 9.73 ± 1.64 nm in size and exhibits a spherical shape. The CQDs showed an antibacterial activity of over 99.9% against *E. coli* at the lowest concentration of 0.75 mg/mL. The antioxidant effects on DPPH exceeded 90% when the CQD concentrations were above 40 µg/mL. This study offers unique insights into a novel method for upcycling and transforming lignocellulosic byproducts into high-value-added multifunctional CQDs.

## Data Availability

The original contributions presented in the study are included in the article, and further inquiries can be directed to the corresponding author.
